# Study protocol: exploring the efficacy of cyclophosphamide added to corticosteroids for treating acute exacerbation of idiopathic pulmonary fibrosis; a randomized double-blind, placebo-controlled, multi-center phase III trial (EXAFIP)

**DOI:** 10.1186/s12890-019-0830-x

**Published:** 2019-04-11

**Authors:** Jean-Marc Naccache, Melissa Montil, Jacques Cadranel, Marine Cachanado, Vincent Cottin, Bruno Crestani, Dominique Valeyre, Benoit Wallaert, Tabassome Simon, Hilario Nunes

**Affiliations:** 1 Assistance Publique – Hôpitaux de Paris (AP-HP), Hôpital Tenon, Service de pneumologie, Site constitutif du centre de référence des maladies pulmonaires rares OrphaLung, Paris, France; 2 Unité de recherche clinique de l’est parisien (URCEst-CRCEst-CRB), Hôpital S Antoine, Hôpitaux Universitaires Paris Est (GH HUEP), Assistance Publique – Hôpitaux de Paris (AP-HP), Paris, France; 3 Centre national de référence des maladies pulmonaires rares OrphaLung, pneumologie, hôpital Louis Pradel, Hospices civils de Lyon, UMR 754, Université Claude Bernard Lyon 1, Lyon, France; 4 Assistance Publique – Hôpitaux de Paris (AP-HP), Hôpital Bichat, Service de Pneumologie A, Site constitutif du centre de référence des maladies pulmonaires rares OrphaLung, Paris, France; 5 INSERM, UMR 1152, DHU FIRE, Université Paris Diderot, Paris, France; 6 Assistance Publique – Hôpitaux de Paris (AP-HP), Hôpital Avicenne, Service de pneumologie, Site constitutif du centre de référence des maladies pulmonaires rares OrphaLung, EA2363, Université Paris, 13 Bobigny, France; 7 CHU Lille, Hôpital Calmette, Service de pneumologie et immunoallergologie, Site constitutif du centre de référence des maladies pulmonaires rares OrphaLung, 59037 Lille, France

**Keywords:** Idiopathic pulmonary fibrosis, Acute exacerbation, Cyclophosphamide

## Abstract

**Background:**

Idiopathic pulmonary fibrosis (IPF) is a fatal lung disease, with a median survival of 2–3 years and variable natural history, characterized by gradual and progressive deterioration. Acute exacerbation of idiopathic pulmonary fibrosis (AE-IPF) is a severe complication, associated with poor survival and a mortality > 50%. To date, no treatment has proven effective in AE-IPF, with cyclophosphamide (CYC) the only therapy suggested to be effective on survival, primarily based on retrospective series. Considering the high fatality rates of AE-IPF, evaluating the efficacy of immunosuppressive agents in a randomized controlled trial proves crucial, as the results could significantly impact treatment and prognosis of AE-IPF.

**Methods:**

The EXAFIP study is a French national multicenter double-blind placebo-controlled randomized trial. Its primary objective is to evaluate the efficacy of CYC compared to placebo on early survival in patients treated with corticosteroids. We hypothesize that adding CYC to high-dose corticosteroids would reduce 3-month mortality in AE-IPF patients. The primary outcome is all-cause mortality rate at Month 3; secondary objectives are to evaluate the efficacy of CYC compared to placebo on overall survival at Months 6 and 12, respiratory disease-specific mortality, respiratory morbidity, and chest high-resolution computed tomography features, and to determine prognostic factors in AE-IPF and compare the safety of the two treatment arms during 6 months’ follow-up.

**Discussion:**

There is an urgent unmet clinical need for effective AE-IPF treatment. The EXAFIP study is the first large Phase III placebo-controlled randomized trial evaluating the efficacy and safety of CYC added to corticosteroids in treating AE-IPF. The results of this study could significantly impact treatment strategy and prognosis of AE-IPF.

**Trial registration:**

Clinical trials, NCT02460588; Date: June 2, 2015, prospectively; Issue date: 14/11/2017; Protocole Amendment Number: 03.

## Background

Idiopathic pulmonary fibrosis (IPF) is a rare disease, primarily occurring from age 60 years onwards, with an estimated prevalence of 1.25–23.4/100.000 and an estimated incidence of 0.22–7.8/100.000 per year in Europe [1]. IPF is a fatal disease with a median survival of 2–3 years. Although usually gradual and progressive, it has been described as presenting with variable history, including acute exacerbations of idiopathic pulmonary fibrosis (AE-IPF) that may prove fatal.

**Fig. 1:**
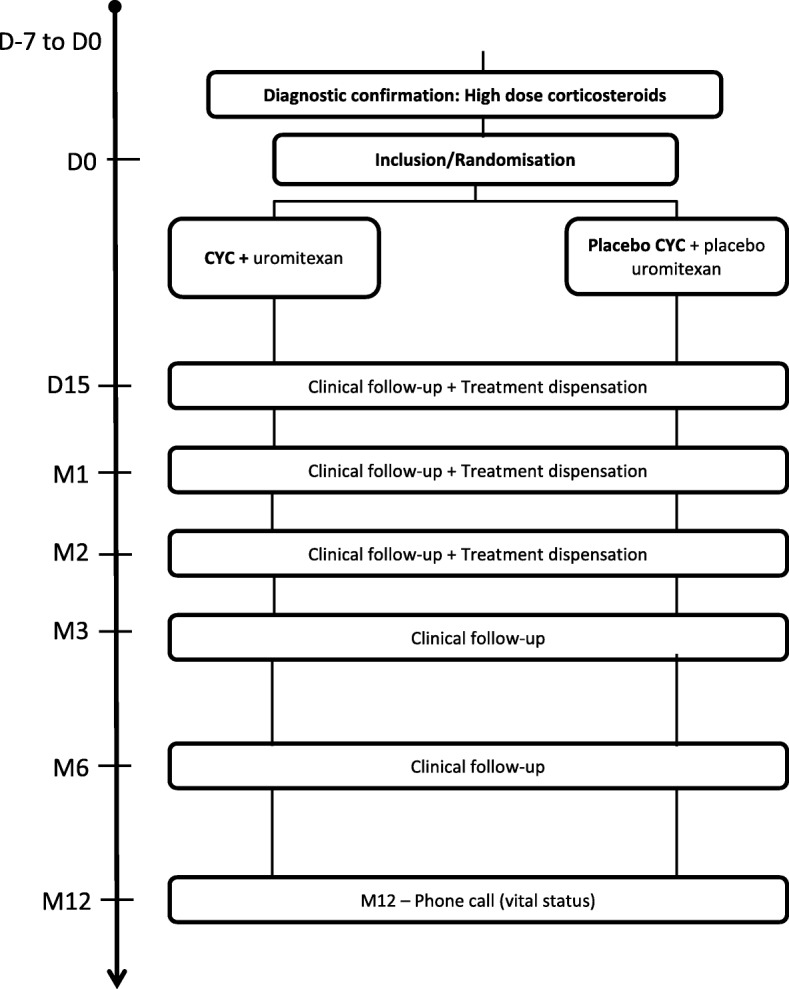
Study design

IPF is a fibroproliferative, irreversible disease of unknown origin. Its pathogenesis comprises initial stretch injury at epithelial-mesenchymal interfaces involving epithelial cell apoptosis, aberrant wound repair by fibroblast accumulation, and extracellular matrix deposition [2]. Inflammation is not considered a main disease driver, as evidenced by the results of the PANTHER randomized trial, which demonstrated that a combination of corticosteroids, azathioprine, and N-acetylcysteine worsened the survival of IPF patients compared to placebo [3].

AE-IPF is considered to be a major event in IPF with an annual incidence of 5–10%, representing the most frequent cause of deterioration requiring hospitalization and accounting for over 30% of patient deaths [4]. AE-IPF is associated with poor outcomes, with an overall 3-month mortality exceeding 50% and reaching up to 90–100% in patients requiring ventilation [5, 6]. AE-IPF pathogenic mechanisms differ from those of stable IPF. Inflammatory pulmonary response constitutes a hallmark of AE-IPF, suggesting the potential efficacy of immunosuppressive therapy.

To date, no treatment has been proven effective in AE-IPF, and current guidelines support using high-dose corticosteroid therapy, such as methylprednisolone pulse dosing [7–12]. Several research groups, however, advocate administering other immunosuppressive therapies, especially cyclosphosphamide (CYC), as either first-line or rescue therapy when corticosteroids fail [7–11, 13–17]. Two French cohort studies focused on AE-IPF suggested that CYC could improve the prognosis of AE-IPF. In the first retrospective study (*n* = 10), the 3-month mortality rate with CYC and methylprednisolone pulse was 45% [7]. In the second study, 3-month mortality rates were 33 and 55% with and without CYC, respectively [8]. More recently, an Italian cohort study evaluating the mortality rate of AE-IPF patients treated with three methylprednisolone pulses followed by a median of five monthly CYC pulse doses reported a 3-month mortality rate of 27% (*n* = 11) [18]. While these series were small-sized, retrospective, and uncontrolled, they do suggest CYC efficacy on survival. Considering the high fatality rates of AE-IPF, evaluating the efficacy of immunosuppressive therapy in this setting by means of a randomized controlled trial proves crucial, as its outcome may significantly impact both treatment and prognosis of AE-IPF.

We hypothesize that adding CYC to high-dose corticosteroids could improve 3-month survival in patients with AE-IPF.

## Methods/design

The EXAFIP study is a French national multicenter (*n* = 34), double-blind, placebo-controlled, randomized trial comparing CYC to placebo in patients receiving high-dose corticosteroids for AE-IPF in terms of early survival at 3 months and overall survival at 12 months.

The total study duration is 42 months, with 120 participants to be included over 30 months.

### Experimental design

The study comprises a screening period (1 week), treatment period (2 months), clinical follow-up (6 months), and telephone follow-up at 1 year for vital status assessment. Patient who withdraw from the study or are non-adherent to protocol will be contacted by telephone at 3, 6 and 12 months to obtain vital status (Fig. 1).

The study coordinator should make at least three attempts to contact the patient or the patient’s emergency contact in a period of 10 days at different time of the day. In the absence of answer, vital status will be collected via contact of the town council of the patient birthplace. Six follow-up consultations are scheduled: Day 0 (inclusion and start of treatment), Day 15, Month (M) 1, 2, 3, and 6.

Screening occurs in the week preceding the inclusion consultation for AE-IPF patients; after diagnosis confirmation, corticosteroid therapy is initiated without delay. The inclusion consultation occurs between Days 1 and 4 following corticosteroid initiation. On inclusion, patients will be randomized and assigned to receive either experimental treatment or placebo.

Dispense of treatment is performed at hospital. Therefore, there is no uncertainty concerning compliance assessment. Data collection plans to record, if at all, the perfusion has been administred and, if not, the proportion that has been.

- In the experimental group, patients will receive CYC and uromitexan (for hemorrhagic cystitis prophylaxis) associated with high-dose corticosteroids.
- High-dose corticosteroids consist of intravenous methylprednisolone, 10 mg/Kg/d (without exceeding 1000 mg) 3 days in a row followed by a shift to 1 mg/Kg/d prednisone for 1 week and 0.75 mg/Kg/d for 1 week, then 0 .5mg/Kg/d for 1 week and 0.25 mg/Kg/d for 1 week, and lastly, 10 mg/d for patients weighing >65Kg vs. 7 .5mg for ≤65Kg until M6.
- CYC administration regimen has been chosen based on our clinical practice in the management of vasculitis cases: 600 mg/m^2^ intravenous CYC (adapted for age and renal function, maximum dose = 1 .2g) at Day 0, Day 15, M1, and M2; 200 mg/m^2^ IV uromitexan at hour (H) 0 and H4, at Day 0, Day 15, M1, and M2.

The CYC dose was adapted according to the following factors: age < 70 years and normal renal function: 0 .6g/m^2^; age ≥ 70 years and normal renal function: 0.5 g/m^2^; impaired renal function (creatinine clearance < 25 mL/min calculated using modification of diet in renal disease [MDRD]): 1/3 dose reduction; neutrophils > 1.5 G/L: 0.6 g/m^2^; neutrophils 1–1.5 G/L: 25% reduction dose; neutrophils <1G/L: 1 week treatment delay.

- In the control group, patients will receive CYC placebo and uromitexan placebo, associated with high-dose corticosteroids.
- In H0, the CYC and intravenous uromitexan placebo consists of 250 mL of NaCl perfused at 0.9% during 90 min. In H4, the uromitexan placebo consists of 100 mL of NaCl perfused at 0.9% during 10 min.

The prohibited concomitant medications are: live attenuated conventional vaccine, phenytoine, vaccination again yellow fever. Pirfenidone and nintedanib are authorized.

### Blinding and treatment allocation

The centralized-blocked randomization is prepared by the clinical research Unit URC-EST, with subject allocation to treatment group performed using a permuted block randomization stratified according to severe IPF (one or more of the following factors: forced vital capacity [FVC] < 50%, diffuse capacity for carbon monoxide [DLCO] < 35%) or not. An independent statistician generates the allocation sequence. The investigators enroll and randomize participants.

Labeling is carried out by the clinical trial department (DEC) of AGEPS (*Agence Générale des Equipements et Produits de Santé*). The treatments are sent to the hospital pharmacies by the DEC of AP-HP AGEPS. The hospital pharmacies are responsible for preparing, blinding, labeling, and dispensing the investigational drugs.

#### Patients are randomized by Internet using the e-CRF

Individual patient treatment assignments will not be unblinded during the study duration unless patient safety issues arise for which unblinding proves necessary in order to ensure optimal patient management. Blinding methods and provisions put in place to maintain blindness.

### Biological sample collection

We take advantage of this study to create a blood mononuclear cell DNA and RNA biobank that should enable us to further investigate the mechanisms underlying AE-IPF. This sample of 120 AE-IPF patients offers a unique opportunity for studying the pathophysiology of this rare disease, providing a demonstration of immunological dysregulation that might lead to targeted therapy with less adverse effects.

### Criteria for discontinuing or modifying allocated interventions for a given trial participant

The investigator can temporarily or permanently end a subject’s participation in the research for any reason that affects the subject’s safety or which would be in the subject’s best interests.

If a patient is lost to follow up, action should be made to know the vital status of the patient. If a subject leaves the research prematurely, data relating to the subject can be used unless an objection was recorded when the subject signed the consent form.

If consent is withdrawn, no data about the subject may be used unless the subject states in writing that he/she does not object. In practice, the subject is excluded from the research.

### Selection of participants

Patients are recruited in respiratory units and intensive care units, and will be included if presenting with definite or suspected AE-IPF, as defined by IPFnet [6].

The diagnostic criteria of AE-IPF are the following: 1) previous or concurrent diagnosis of IPF; 2) unexplained exacerbation or development of dyspnea within 30 days; 3) high-resolution computed tomography (HRCT) revealing new bilateral ground-glass abnormalities or consolidation superimposed on a IPF background; 4) no evidence of active pulmonary infection based on endotracheal aspirate or bronchoalveolar lavage, if feasible; 5) exclusion of alternative causes, such as left-heart failure, pulmonary embolism, and identifiable causes of acute lung injury.

Patients who fail to meet all five criteria due to missing data, especially HRCT data, are considered “suspected AE”. Regarding the revised diagnostic criteria, both idiopathic and triggered AE will be considered [19].

Inclusion criteria are as follows:

- ≥18 years of age;
- Definite or probable IPF diagnosis, based on 2011 international recommendations (6);
- Definite or suspected AE, as defined by IPFnet [6] criteria after excluding alternative diagnoses for acute worsening;
- Efficient contraceptive method initiated within 1 month for women and 3 months for men;
- Social security coverage;
- Ability to understand and sign a written informed consent form.

Non-inclusion criteria are as follows:

- Identified etiology for acute worsening status (i.e., infectious disease);
- Known hypersensitivity or contra-indication to either CYC or any component of the study treatment;
- Mechanical ventilation;
- Active bacterial, viral, fungal, or parasitic infection. On swab testing, only positive results for Influenza A, Influenza B, and Respiratory Syncytial Virus (RSV) are considered active viral infections;
- Active cancer;
- Registered on a lung transplantation waiting list;
- CYC treatment received in the previous 12 months;
- Participation in another clinical trial;
- Documented pregnancy or breast-feeding.

### Trial objectives and outcomes

The primary objective of this study is to evaluate the efficacy of CYC compared to placebo on early survival (3 months) in patients treated with corticosteroids.

The secondary objectives are to evaluate the efficacy of CYC compared to placebo on overall survival, respiratory disease-specific mortality, respiratory morbidity, and chest HRCT features in patients treated by corticosteroids, to determine prognostic factors in AE-IPF and to compare the safety of the two treatment arms.

The primary outcome is all-cause mortality rate at M3. This choice as primary assessment criterion is based on previous data obtained from retrospective series.

Secondary efficacy outcome variables are as follows:

- Overall survival at M6 and M12;
- Mortality related to respiratory disease at M3 and M6;
- Prognosis factors of AE-IPF mortality: PFT results before AE-IPF, time until consultation after clinical worsening, laboratory evaluation (LDH, CRP) at AE-IPF diagnosis, PaO^2^ at AE-IPF diagnosis, chest HRCT features at AE-IPF diagnosis, time until receiving treatment for AE-IPF;
- Respiratory morbidity, defined as any of the events listed below and occurring between M0 and M6:
- Worsening dyspnea (0–100-mm visual analogue [VAS] scale anchored with 0 = “no breathlessness” and 10 or 100=“worst imaginable breathlessness”. Worsening is defined as an absolute decrease of 10 mm);
- Or increased need for oxygen of over 3 L/min to obtain SaO2 > 90% or decrease in PaO^2^ of over 10 mmHg with the same rate of supplemental oxygen flow;
- Or decrease in FVC of over 10% of the predicted value;
- Or decrease in DLCO of over 15% of the predicted value.
- Chest HRCT features at M3 and M6 compared to inclusion;
- Safety outcome measures between M0 and M6: clinical laboratory evaluation (blood cell count, serum creatinine measurement) according to the Common Terminology Criteria for Adverse Events (CTCAE), hemorrhagic cystitis (occurrence of hematuria on urine dipstick and pelvic pain or dysuria indicating need for cystoscopy), number of infectious diseases, diabetes mellitus (capillary blood glucose monitoring and fasting plasma glucose > 1. 26 g/L), and hypertension (blood pressure > 160/100 mmHg).
- All suspected unexpected serious adverse reactions are declared by the sponsor, within the legal time frame, to the *Agence Française de Sécurité Sanitaire des Produits de Santé* (ANSM, French Health Products Safety Agency) and to the relevant *Comité de Protection des Personnes* (CPP, ethical committee). Once a year for the duration of the clinical trial, the sponsor must draw up an annual safety report

#### Specific research committees

##### Steering committee

The missions of the steering committee are as follows: 1) to define the research objectives; 2) to propose changes to the protocol during research, as necessary; 3) to organize the research, determine its methodology, coordinate the information made available, and monitor the research conduct.

Amendments are submitted to both the ethical committee and French Drug Safety Agency for approbation.

##### Data safety monitoring board (DSMB)

The members of DSMB are independent of the research, with at least one member having expertise in ILD. The DSMB missions are as follows: to review safety data at regular intervals, to assess the need for prematurely stopping the research, to analyze the causes of deaths, as well as important changes to the protocol. Any substantial modification to the protocol by the coordinating investigator must be sent to the sponsor for approval.

### Data management and confidentiality

Data will be collected using an electronic case report form, designed by the study coordinator in collaboration with URC-EST.

Data will be completed by the investigators at each follow-up visit with the help of a Clinical Research Technician (CRT) of the URC-Est for AP-HP centers.. Data entry will be monitored by a Clinical Research Assistant (CRA) and will be checked for missing values and consistency by a data manager. These controls are described in a data validation plan.

Thoe people responsible for biomedical research quality control will take all necessary precautions to ensure the confidentiality of information about the experimental medications, research, research subjects, and particularly, the identity of the subjects and results obtained. During or after the biomedical research, the data collected on the research subjects and sent to the sponsor by the investigators will be anonymized.

All data, documents, and reports may be subject to regulatory audits and inspections. An audit can be carried out at any time by individuals appointed by the sponsor and who are not associated with the research directors.

### Statistical analyses

#### Sample size

Reviewing all the series reported between 1993 and 2006 revealed a 3-month mortality rate of 67.1% (95% confidence interval [CI]: 56.2–76.5) [5].

We thus hypothesize that the 3-month mortality rate with methylprednisolone pulse dosing will be 67%, decreasing to 42% after adding CYC. Given that alpha = 5%, power = 80%, and two-sided tests will be applied, 60 subjects per group are needed. In case of dropouts, additional patients will be included within the limit of 10% of the initial number of patients needed.

Therefore, 120 patients should be recruited in a period of 24 months and that correspond to 13% of the expected new patients.

#### Primary and secondary outcome analysis

The baseline patient characteristics will be described according to the treatment group.

Qualitative data will be expressed using frequencies and percentages, and quantitative data using either means and standard errors or medians, interquartile ranges, and ranges, as appropriate.

The “center” factor will not be considered in statistical models due to the high number of participating centers and the anticipated small number of subjects randomized in each.

Analyses will be conducted based on the intent-to-treat (ITT) population. All-cause mortality rate at M3 will be compared between groups using Chi-squared or Fisher’s exact tests. In case of missing data on principal criteria, the missing = failure method will be applied as replacement measure. To test the robustness of the analyses, analyses under bias maximum hypothesis will be realized. No replacement of other missing data is planned.

The study aims to compare overall survival (time from randomization until death) between groups at M6 and M12. Premature discontinuation of research will be censored at the last follow-up visit available, while premature discontinuation of treatment will not be censored.

M3 and M6 Kaplan-Meier estimates and confidence intervals will be tabulated. Greenwood’s variance estimate will be used to calculate two-sided 95% CIs. Groups will be compared in univariate analysis by log-rank test.

A Cox’s proportional hazards model will be created to assess treatment efficacy, considering treatment discontinuation as a time-dependent covariate.

Mortality related to the respiratory disease at M3 and M6 and respiratory morbidity will be compared between groups by Chi-squared or Fisher’s exact tests.

Morbidity at M6 will be described and compared between groups using Chi-squared or Fisher’s exact tests. Variation in the global extent of interstitial features on HRCT between M0, M3, and M6 will be compared between groups by *t*-test or Mann-Whitney/Wilcoxon test, as appropriate.

For each evaluation time point, clinical laboratory evaluation will be expressed as means and standard errors or medians, interquartile ranges, and ranges. Boxplots will be created for graphic representation. Percentages of abnormal clinical laboratory results (blood count, serum creatinine measurement), hemorrhagic cystitis (urine dipstick), infectious disease, diabetes mellitus (capillary blood glucose monitoring), and hypertension will be compared between groups using Chi-squared or Fisher’s exact tests. No interim analysis data planned.

## Discussion

IPF is a devastating disease with a median survival time of 3–4 years [20]. AE-IPF constitutes a significant event occuring in the natural history of IPF with a high mortality rate. At present, patients suffering from AE-IPF are administered systemic corticosteroids, but no treatment has been clearly proven effective.

To better reflect the current state of knowledge regarding AE-IPF and improve the feasibility of future research into its etiology and treatment, the International Working Group Report iteratively developed in 2016 a new conceptual framework for acute respiratory deterioration in IPF and revised the definition and diagnostic criteria for AE-IPF [19]. This article provides a short literature review on all the studies published since 2007 and emphasizes that, while there have been many articles describing various potential therapies for AE-IPF published over the last decade, these studies were mostly small-sized and uncontrolled [19]. Concerning CYC therapy, approximately 128 cases were reported, yet without any control subjects [10, 14, 21, 22]. Many studies have suggested that combining an immunosuppressant (cyclosporine, cyclophosphamide, or tacrolimus) with corticosteroids proves likely more effective than corticosteroid alone, with better survival obtained with this option. These studies, however, included only small patient numbers, and all were uncontrolled [23–25].

### Rationale for conducting the trial

The EXAFIP study is the first large Phase III trial evaluating CYC therapy in patients with AE-IPF. In this patient population, the pathophysiology likely involves an exaggerated inflammatory response, whether either idiopathic or triggered. In most cases, the natural history of AE-IPF proves fatal, in spite of corticosteroid therapy (not approved). Cyclophosphamide is available for interstitial lung disease (ILD) associated with scleroderma, and for acute and severe ILD associated with myositis [26, 27]. In contrast to ILD associated with scleroderma, no prospective, controlled clinical trial has been performed in other ILD forms, probably due to their relative rarity. Therefore, we suggest that combined intravenous pulse doses of high-dose corticosteroids and CYC could represent a reasonable add-on therapy for AE-IPF. However, this therapeutic strategy requires to be tested in controlled studies and this, not only to confirm its beneficial impact on AE-IPF prognosis but also to avoid any unsuspected deleterious effects, as previously observed with immunosuppressors in stable IPF. Large networks of patients and clinicians will be needed to assess the efficacy of therapeutic interventions, while taking into account the influence of confounding factors considering the disease’s low prevalence and the fact that the studies can only be conducted in patients presenting with confirmed episodes of AE-IPF.

### Rationale for patient selection

The protocol criteria for patient selection mirrors the physician’s assessment in routine clinical practice, as both are based on AE-IPF diagnosis criteria. Suspected AE-IPF is defined as an idiopathic acute respiratory worsening status that cannot be classified as a definite acute exacerbation due to missing data or criteria. Critically ill patients are not always able to undergo the examinations required for establishing a definite AE-IPF diagnosis. Our decision to include suspected AE-IPF in EXAFIP was based on a previous study that demonstrated that suspected AE-IPF is clinically indistinguishable from definite acute exacerbation, with a similar prognosis [28]. Though chosen before the publication of revised definition of AE-IPF, these criteria are in accordance with the new AE-IPF criteria [19].

### Rationale for study endpoints

The study endpoints are consistent with previous retrospective AE-IPF series. The use of 3-month mortality as a primary endpoint is a robust criterion with minimum missing data. Overall survival at M6 and M12 and mortality related to respiratory disease at M3 and M6 as secondary endpoints offer a similar advantage despite being more difficult to analyze. EXAFIP should enable prospective data about respiratory morbidity, prognosis factors, and HRCT features in AE-IPF to be made available for the first time in the disease history. Safety outcomes are likewise paramount, considering the results of previous studies focused on promising drugs, reporting that immunosuppressive agents and antivitamin K were proven deleterious in IPF [29, 30]

## Conclusion

There is currently no proven beneficial treatment for AE-IPF patients, and therefore no evidence-based management strategy to be applied, hence the urgent unmet clinical need for effective therapy for this patient population. The EXAFIP study is the first placebo-controlled randomized trial evaluating the efficacy and safety of CYC added to corticosteroids in the treatment of AE-IPF.

If the study demonstrates efficacy, this could have a major impact on the treatment strategy for AE-IPF and improve these patients’ prognoses.

## Abbreviations

AE: Acute exacerbation
AE-IPF: Acute exacerbation of idiopathic pulmonary fibrosis
AGEPS: Agence Générale des Equipements et Produits de Santé
AP-HP: Assistance Publique – Hôpitaux de Paris
CTCAE: Common Terminology Criteria for Adverse Events
CYC: Cyclophosphamide
DEC: Clinical trial department
DLCO: Diffusing capacity of the lungs for carbon monoxide
FVC: Forced vital capacity
HRCT: High-resolution computed tomography
ILD: Interstitial lung disease
IPF: Idiopathic pulmonary fibrosis
ITT: Intent-to-treat
MDRD: Modification of diet in renal disease
VAS: Visual analogue scale
